# Power calculations for cluster randomized trials (CRTs) with right-truncated Poisson-distributed outcomes: a motivating example from a malaria vector control trial

**DOI:** 10.1093/ije/dyz277

**Published:** 2020-02-03

**Authors:** Lazaro M Mwandigha, Keith J Fraser, Amy Racine-Poon, Mohamad-Samer Mouksassi, Azra C Ghani

**Affiliations:** 1 Department of Infectious Disease Epidemiology, MRC Centre for Global Infectious Disease Analysis, Imperial College London, London, UK; 2 Department of Statistical Methodology and Consulting, Novartis Pharma AG, Basel, Switzerland; 3 Bill & Melinda Gates Foundation, Seattle, WA, USA; 4 Department of Strategic Consulting Integrated Drug Development, Certara, Montreal, QC, Canada; 5 School of Pharmacy, Department of Pharmaceutical Sciences, Lebanese American University, Byblos, Lebanon; 6 Faculty of Pharmacy, Department of Pharmaceutical Sciences, University of Montreal, Montreal, QC, Canada

**Keywords:** Truncated outcomes, statistical power, sample size, vector control trial

## Abstract

**Background:**

Cluster randomized trials (CRTs) are increasingly used to study the efficacy of interventions targeted at the population level. Formulae exist to calculate sample sizes for CRTs, but they assume that the domain of the outcomes being considered covers the full range of values of the considered distribution. This assumption is frequently incorrect in epidemiological trials in which counts of infection episodes are right-truncated due to practical constraints on the number of times a person can be tested.

**Methods:**

Motivated by a malaria vector control trial with right-truncated Poisson-distributed outcomes, we investigated the effect of right-truncation on power using Monte Carlo simulations.

**Results:**

The results demonstrate that the adverse impact of right-truncation is directly proportional to the magnitude of the event rate, *λ,* with calculations of power being overestimated in instances where right-truncation was not accounted for. The severity of the adverse impact of right-truncation on power was more pronounced when the number of clusters was ≤30 but decreased the further the right-truncation point was from zero.

**Conclusions:**

Potential right-truncation should always be accounted for in the calculation of sample size requirements at the study design stage.

**Key Messages**

- Right-truncation attenuates (statistical) power.
- This attenuation is more pronounced when the numbers of clusters is less than or equal to 30 and when the point of truncation is closer to lower bound of the Poisson distribution.
- Closed-form formulae for sample size requirements for cluster randomized trials (CRTs) are not appropriate for right-truncated Poisson-distributed outcomes.
- Sample size calculations for CRTs with right-truncated Poisson-distributed outcomes should include a correction to the probability mass function (PMF) of the Poisson distribution.

## Background

Cluster randomized trials (CRTs) are trials in which randomization of the intervention under study is applied to groups. These groups are referred to as clusters and may consist of individuals with shared characteristics.^1^ Thus, outcomes within a cluster are expected to be correlated.^2^ CRTs are frequently used in epidemiology to the evaluate the efficacy of interventions targeted at the population level (see for example Hayes *et al*.^1^). One specific area of application of CRTs within epidemiology is the control of vector-borne diseases (VBD)—in which infection passes between vectors and human hosts. For such diseases, interventions frequently target the vector (through, for example, the application of insecticides) whereas the outcome of interest is infection or disease in the human host. Since such tools are implemented at the level of a cluster, their efficacy can only be evaluated through CRTs.^3^^,^^4^

The design of CRTs requires prior calculation of sample sizes that would be sufficient to determine the efficacy of the vector control intervention (often referred to as ‘power calculations’). For CRTs, the required sample size is a function of the number of clusters, the corresponding cluster sizes and the between-cluster variance for which the desired power is achieved.^2^ The between-cluster variance contributes to the computation of either the intracluster correlation (ICC) or the coefficient of variation (CV) which quantify the magnitude of similarity (correlation) in the outcome within clusters.^5^ For continuous and binary outcomes, the ICC (typically denoted by ρ) is defined as the ratio of the between-cluster variance to the total variance (both within and between the clusters^6^^,^^7^). On the other hand, the CV, denoted by *k*, is defined as the ratio of the between-cluster standard deviation to the parameter of interest (e.g. mean, proportion or rate) within each cluster.^8^ Therefore, the between-cluster variance is typically accounted for by incorporating ρ or *k* in the closed-form sample size calculation formulae for CRT designs.^5^^,^^8^ It is only in the case of binary outcomes that the CV may be easily converted to the ICC (and vice versa).^7^^,^^9^ Generally, an increase in ρ or *k* leads to a corresponding increase in the number of clusters and/or cluster sizes required to achieve the desired power, as the corresponding increase in between-cluster variance results in decreased precision in the estimates of parameters of interest.^8^^,^^10^

The closed-form formulae for calculating sample sizes for the desired power for CRTs vary for different types of outcomes (e.g. normal, binary, time to event, Poisson etc.) and study designs (such as cross-over, stepped-wedge, matched designs etc.). These formulae assume that the domain of the outcomes being considered (whether continuous, binary, ordinal, count etc.) covers the full range of values of the considered distribution as defined by the population parameters. However, in many epidemiological trials where the outcome under consideration is the number of times a host tests positive for an infection over a specified period, there are practical limits to the number of times a person may be tested for the disease, introducing truncation into this distribution. Thus, the computation of sample size requirements from existing formulae in such instances may result in incorrect estimates of power^5^^,^^8^^,^^11–13^ and inconsistent parameter estimates from subsequent statistical analysis of the trial data.^14^ Using a motivating example from the design of a new vector-based intervention for malaria, we investigate the consequences of truncation on the calculations of statistical power.

## Methods

### Motivating example—vector control trials for malaria

Vector control tools (VCTs) are an integral part of control for malaria. We consider an application to a new tool currently under consideration—attractive targeted sugar baits (ATSB)^15^^,^^16^—which kill male and female mosquitoes after feeding on synthetic baits. In doing so, ATSBs reduce the overall mosquito population and additionally reduce the probability that mosquitoes survive sufficiently long to transmit infection.^17–19^ This results in a reduction in the total and infectious mosquito population (entomological endpoints) which consequently is expected to result in a reduction in malaria prevalence and clinical incidence in humans (epidemiological endpoints).

The efficacy of new VCTs for malaria is generally assessed in CRTs with randomization conducted at the village level. However, epidemiological endpoints are generally not assessed for the whole village, but rather within a nested cohort. There are several reasons for this. First, children are at higher risk of disease and detectable infection than adults who develop partial immunity with continued exposure, hence power can be improved by focusing on children.^20^ Second, it is not ethically acceptable to detect clinical malaria in a trial participant without providing treatment—which in high transmission areas could result in a large number of villagers receiving treatment and therefore modifying onward transmission and thus biasing the trial. Third, the cost of follow-up across the whole village can become significant, and thus it is often more convenient and cost-effective to recruit a smaller cohort.

**Figure 1:**
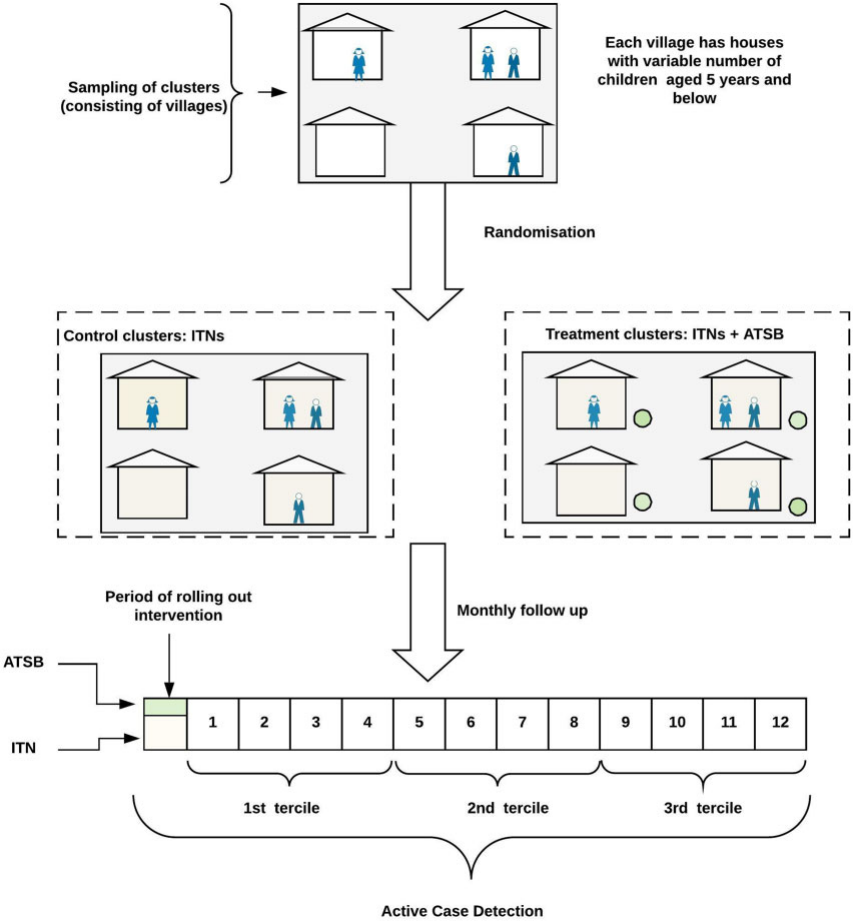
Conceptual design for the attractive targeted sugar baits (ATSB) trial. The treatment clusters have ATSB feeding stations outside the houses (denoted by the green circle) in addition to insecticide-treated nets (ITNs) inside each house. The control clusters have only ITNs in the houses, the standard of care recommended by the World Health Organization (WHO). Therefore, the effect size is the efficacy of the ATSB detectable over and above that of ITNs.

A typical design for a vector control trial is illustrated in Figure 1. A subset of children in the intervention and control villages are recruited at the beginning of the trial and their infections are cleared. They are then followed using active case detection (either for clinical disease or presence of infection), typically at 1-monthly intervals. The epidemiological outcome is therefore the count of monthly malaria episodes over the trial period. For most trials, children are recruited in a single cohort and followed up over the entire period of the trial (1 year). However, to overcome cohort fatigue and reduce drop-out, an alternative design proposed is to recruit multiple cohorts of children sequentially three times, with each cohort followed for 4 months (a tercile). The rationale for recruiting multiple cohorts is that a single cohort followed up over a long period of time may be characterized by high drop-out compared with multiple cohorts followed up over a shorter period (tercile). Altogether, the terciles comprise a 1-year trial period.

In areas of moderate to high transmission typical of malaria in Africa, children experience on average one to three episodes of clinical malaria a year, with several experiencing five or more and some experiencing more than 10.^21^ However, the design of the trial can act to truncate the upper values of this distribution. In our example above, we can theoretically observe a maximum of 12 malaria episodes per child during a 1-year trial because active detection occurs monthly. In practice, the detected number of episodes is likely to be fewer since: (i) malaria is often seasonal and so episodes will be concentrated within a 4–6 month period; and (ii) following a detected malaria episode (which may not have been detected in the absence of the study), treatment will provide a period of protection against re-infection of up to 25 days.^22^^,^^23^ Thus, under this design it is reasonable to expect to detect a maximum of six malaria episodes per child per year in the first setting and a maximum of two in every tercile in the second setting.^21^

### A model for cluster-randomized trial outcomes

We let Y denote the count of malaria episodes in a year with $Y∼\mathrm{Poisson}\left(\lambda \right)$. Let the event rate with a log-link be denoted by:

(1) $$\ln\left(\lambda \right)=\beta_{o}+{\;\beta }_{1}x+c_{i}$$

where $\beta_{o}\;$and ${\;\beta }_{1}$ are the fixed effects representing the log rate of the control and the intervention effect, respectively, *x* denotes the allocation of the village to either control (*x *=* *0) or intervention (*x *=* *1) and $c_{i}\;∼N(0,\;\sigma_{c}^{\;2})$ is a random effect that models cluster-specific predictions for each of the clusters *i *=* *1*,* 2 *… n_c_*^24^ where the term $\sigma_{c}^{\;2}$ denotes the between cluster-variance used to compute ICC. Unlike for continuous and binary outcomes, the ICC for count outcomes is undefined.^25^ However, for count outcomes with equal follow-up time,^10^ Stryhn *et al.*^26^ developed an approximate method of computing the ICC based on model linearization as shown in equation (2). The approximation makes use of the term ($\beta_{o}+{\;\beta }_{1}x)\;$which is the linear predictor from the model in equation (1) as well as $\sigma_{c}^{\;2}$ and λ which represent the between-cluster variance and event rate, respectively.

(2) $$\mathrm{ICC}\approx \frac{\sigma_{c}^{\;2}\;\times \;e^{-2(\beta_{o}+{\;\beta }_{1}x)}}{\left(\{\sigma_{c}^{\;2}\;\times \;e^{-2(\beta_{o}+{\;\beta }_{1}x)}\}+\;\lambda \right)}$$

### Truncated Poisson-distributed outcomes

The probability mass function (PMF) for the (untruncated) Poisson distribution is shown in equation (3) where *Y* denotes the random variable (the count of malaria episodes per child), *λ* denotes the mean count of malaria episodes and *y* denotes the realization of the random variable which can take any positive integer (including zero) and is unbounded from above.

(3) $$P\left(Y=y:\lambda \right)=\frac{e^{-\lambda }\lambda^{y}}{y!},\;y=0,\;1,\;2,\ldots$$

Suppose that realizations of the Poisson distribution are right-truncated at a value denoted by *T* such that 0 ≤ *Y *≤* T*. The PMF then takes the form shown in equation (4), which is equivalent to the product of an untruncated Poisson PMF divided by the cumulative density function (CDF) of the right-truncated Poisson distribution.^27–29^ Note that when T=∞, this implies lack of truncation and thus equation (2) is recovered.

(4) $$P\left(Y=y:\lambda :y\leq T\right)=\frac{e^{-\lambda }\lambda^{y}}{y!}\;{\left\{\sum_{z=0}^{T}\frac{e^{-\lambda }\lambda^{z}}{z!}\right\}}^{-1}$$

### Estimating power for right-truncated Poisson-distributed outcomes

For event rate data, formulae exist in the literature for the estimation of power for CRTs.^5^^,^^11^ These formulae assume that the count outcome is completely defined by the standard Poisson distribution and therefore do not account for (right-) truncation as shown in equation (4). In instances of right-truncation, these closed-form sample size formulae would not be appropriate to use. Therefore, as recommended by Landau *et al*.^30^ in such instances where closed-form formulae are inapplicable, a simulation-based approach to computing power was undertaken. The impact of the degree of truncation was evaluated in three broad settings. First, the number of clusters and the corresponding cluster sizes that would yield ∼80% to 85% statistical power given a specified λ and $\sigma_{c}^{\;2}$ were determined under the assumption that the count events were not right-truncated (T=∞). Second, an extreme case of truncation was simulated by assuming T=1 whereby only a maximum of one event (incidence or presence of clinical disease) would be observed during the trial period. Notice that this particular situation is equivalent to the binary outcome in which the interest is in the presence of infection or clinical disease (Yes = 1, No = 0). This is statistically important because for this special case of right-truncation, formulae for statistical power^5^^,^^8^ and statistical models such as generalized estimating equations (GEEs)^31^ and generalised linear mixed models (GLMMs)^32^ account for binary nature of the outcome and thus this type of right-truncation. However, for other cases of right-truncated event outcomes (where T >1), the impact of truncation on power is seldom considered or dealt with. This formed the third setting of right-truncation considered. Therefore, the PMF in equation (3) for the three settings considered had T=∞, T = 1 and T= t (where t was finite but greater than one), respectively. The annual mean number of malaria episodes, λ =(1.25, 2.7), and ICC [computed from $\sigma_{c}^{\;2}\;(0.05,\;0.1,\;0.2,\;0.3,\;0.4),\;$see equation (2)] for the simulations were all within the range of values informed by past malaria epidemiological studies based on data derived directly or indirectly from sub-Saharan Africa.^33–37^

Specifically, for annual mean number of malaria episodes of 1.25 and 2.7, the selected cohort of children were followed up over 12 months with right-truncations considered at T=∞, 1, 3 and 6. The between-cluster variances were pre-specified at $\sigma_{c}^{\;2}\;(0.05,\;0.1,\;0.2,\;0.3,\;0.4)$. In addition, we considered the effect of cohort switching (every 4 months) on power. For this scenario we assumed a mean of 2.7 episodes per year to explore a region with sufficient power, and considered truncation levels for each 4-month period assuming year-round transmission (T=∞, 1 and 2, respectively). This in effect introduced a new cohort in every tercile of the study (see Figure 1) which was accounted for by introducing an offset term equal to log (4/12 years) in equation (1). In all of the simulations, balanced randomization was conducted (i.e. equal allocation to treatment and control arms). For each of the simulated datasets based on the PMF [equation (3) or (4) as appropriate based on pre-specified values of T], the empirical between-cluster variances were derived from the model fitted (equation (1). These empirical between-cluster variances were tracked across all the datasets (to ensure that the simulations were in keeping with the pre-specified cluster variances) and were also used to compute the empirical ICC as defined by equation (2). When T = ∞, the Poisson outcomes were simulated using the R software function *rpois* and were analysed using the functions *glmer* from the R packages *lme4*^38^ and *lmerTest.*^39^ For other values of T (presence of right-truncation), the Poisson outcomes were simulated using the R function *rtrunc* from the R package *truncdist*^40^^,^^41^ and were analysed using the R function *gnlmm* from the R package *nlmixr.*^42^ The simulations were also replicated in *SAS* using *PROC NLMIXED.*^43^^,^^44^ Hypothesis testing was conducted ($H_{1}$:$\;{\;\beta }_{1}\;\neq \;0$ where ${\;\beta }_{1}<0)\;$with statistical power calculated as the proportion of 1000 samples in which the effect of intervention was detected. The SAS and R code for the simulations are provided as Supplementary files, available as Supplementary data at *IJE* online.

## Results

### Impact of truncation in a cluster randomized trial with a single cohort

Table 1 summarizes the simulated impact of right-truncation on statistical power for the setting where a single cohort was recruited for the entire length of the trial (12 months). The second and third column of the table show the numbers of clusters and corresponding cluster sizes required to achieve a power of approximately 80% when it is assumed that right-truncation is absent (see column where T = ∞). Generally, for any combination of λ and $\sigma_{c}^{\;2}$, the statistical power was highest when no right-truncation was present and lowest when right-truncation engendered a binary outcome (T = 1), with the widest gap between these estimates observed when λ  = 2.7. For other values of T, the discrepancy in the estimates of power from when T = ∞ was large when the number of clusters considered was ≤30, with the largest discrepancy observed for λ  = 2.7. The least discrepancy in power estimated between the untruncated setting and right-truncated setting was observed for T = 6. As expected, an increase in ICC led to an increase in the sample size required to maintain power at 80%.

**Table 1.** Summary of results highlighting the impact of right-truncation (denoted by T = 6, 3 and 1) on the calculations of statistical power over a range of settings where statistical power was initially between 80% and 85% when no truncation was present (T= ∞) for a single cohort recruited over the trial period. The pre-specified between-cluster variance, $\sigma_{c}^{\;2},$ is the value of the between-cluster variance inputted for simulation of the trial data, and the average empirical between-cluster variance is the mean between-cluster variance estimated from the simulated datasets for each combination of λ and $\sigma_{c}^{\;2}$. The average empirical ICC is the mean intracluster correlation computed as described by Stryhn *et al*.^26^ from the simulated datasets

						Statistical power (%)			
	Number of clusters	Cluster sizes	Pre-specified between-cluster variance ($\sigma_{c}^{2}$)	Average empirical between-cluster variance	Average empirical ICC	T = ∞	T = 6	T = 3	T = 1
Annual event rate (λ) = 1.25	30	15	0.050	0.043	0.038	83.4	80.6	73.4	43.3
	30	45	0.100	0.091	0.078	80.0	77.7	75.7	64.2
	60	35	0.200	0.191	0.150	82.7	82.9	82.2	69.4
	90	40	0.300	0.292	0.210	83.5	85.2	83.4	76.6
	110	40	0.400	0.389	0.260	82.6	80.2	79.8	75.8
Annual event rate(λ) = 2.7	25	10	0.050	0.041	0.074	82.5	79.8	56.9	24.8
	30	30	0.100	0.092	0.153	82.4	79.6	73.2	49.6
	55	20	0.200	0.189	0.269	82.7	78.4	74.4	53.7
	80	30	0.300	0.294	0.360	82.6	79.7	76.8	64.6
	110	25	0.400	0.392	0.427	82.9	82.0	79.8	66.4

### Impact of truncation in a cluster randomized trial with multiple cohorts

Table 2 shows the results for λ = 2.7 where three cohorts were recruited over the trial period. Each cohort was followed up over a period of 4 months.

**Table 2.** Summary of results highlighting the impact of right-truncation in every tercile (denoted by T = 1 and 2) on the calculations of statistical power over a range of settings where statistical power was initially between 80% and 85% when no truncation was present (T = ∞) for multiple cohorts recruited over the trial period. The pre-specified between-cluster variance, $\sigma_{c}^{\;2},$ is the value of the between-cluster variance inputted for simulation of the trial data, and the average empirical between-cluster variance is the mean between-cluster variance estimated from the simulated datasets for each combination of λ and $\sigma_{c}^{\;2}$. The average empirical ICC is the mean intracluster correlation computed as described by Stryhn *et al*.^26^ from the simulated datasets

						Statistical power (%)		
	Number of clusters per tercile	Cluster sizes	Pre-specified between-cluster variance ($\sigma_{c}^{2}$)	Average empirical cluster variance	Average empirical ICC	T = ∞ per tercile	T = 2 per tercile	T = 1 per tercile
Annual event rate(λ) = 2.7	25	10	0.050	0.041	0.378	82.5	70.8	44.1
	30	30	0.100	0.092	0.592	82.4	78.5	70.9
	55	20	0.200	0.189	0.751	82.7	76.0	70.9
	80	30	0.300	0.294	0.823	82.6	77.7	75.1
	110	25	0.400	0.392	0.861	82.9	81.8	80.5

Compared with the setting where λ = 2.7 and only a single cohort is followed up over the trial period, the recruitment of multiple cohorts leads to a slight a loss in statistical power. For example, for $\sigma_{c}^{\;2}=0.05$, T = 2 per tercile which is equivalent to T = 6 over the entire trial period had power estimated at 70.8% and 79.8%, respectively. However, the corresponding overall sample sizes involved over the trial period were 25*10*3 = 750 and 25*20 = 250, respectively. As may be seen from the figures, the recruitment of multiple cohorts may result in a substantial increase in the cost of the trial with no corresponding gain in power compared with when a single cohort is recruited. However, in instances where the rate of drop-out is extremely high after 4 months of follow-up due to cohort fatigue, the recruitment of multiple cohorts would turn out to be more cost-effective, as the setting with a single cohort recruited would result in massive loss of power over the 12 months.

## Discussion

Cluster randomized trials are costly but are a critical part of the evidence-gathering framework that is necessary for effective decision making for VCTs.^3^ For them to be cost-effective, they need to be well-designed with appropriate methodology. The estimation of power is critical to ensure that CRTs are of sufficient size to detect the effect of the intervention if it exists. Underpowered CRTs are unlikely to determine the efficacy of VCTs which, as well as wasting the resources committed, could additionally result in effective interventions not being identified. Power calculations for VCTs are therefore recommended irrespective of whether the endpoint is epidemiological or/ and entomological.^3^ Guidelines for the design of robust CRTs including power calculations formulae are well-documented in the literature.^1^^,^^5^^,^^8^^,^^10^ For the specific case of closed-form formulae for the estimation of power for CRTs with Poisson-distributed outcomes,^5^^,^^11^ none of these formulae is appropriate when the count outcome is right-truncated. Therefore, for right-truncated Poisson-distributed outcomes, power calculations for CRTs may be conducted using Monte Carlo simulations.^30^

Our results, based on Monte Carlo simulations, show that for any combination of the event rate, λ, and ICC, statistical power was highest when right-truncation was absent. Right-truncation had an attenuating effect on power, with the lowest power observed when right-truncation resulted in the binomial situation of a maximum of one event (T = 1 over the trial period) and when the number of clusters considered was less than or equal to 30. The adverse effect of right-truncation was less pronounced as settings moved away from binomial situation (i.e. T >1), with situations where T = 6 resulting in modest discrepancies in power compared with when truncation was absent. Moreover, the adverse impact of truncation for any right-truncation value (T) was more pronounced for λ = 2.7 than for λ = 1.25, suggesting that the impact of right-truncation worsens with increased rate of events especially where the difference in value between λ and T is small. This means for instance that for T = 3, the impact of right-truncation will be worse for λ = 2.7 than for λ = 1.25, because a significant portion of the distribution is ‘cut out’ for higher rates.

A CRT design which mitigates the impact of high drop-out rates through the recruitment of multiple cohorts was also considered. The results suggest that under such a design, right-truncation has a far more negative impact compared with the design where a single cohort is recruited. This is because the use of multiple cohorts results in shorter follow-up times for each cohort recruited, which further limits the number of events that may be observed. In effect, this inadvertently induces stricter right-truncation scenarios. Thus, compared with the sample size that would be required if a single cohort was recruited for the entire duration of a trial, the use of multiple cohorts increases the sample size required to maintain the targeted level of power. This increase in sample size would lead to adverse cost implications. That said, the recruitment of multiple cohorts may turn out to be cost-effective in instances where the drop-out rate for a recruited cohort is substantial after a few months (4 months for the case considered). Therefore, the impact of multiple cohorts on statistical power needs to be carefully weighed against the drop-out rate at the trial design stage.

This study has several limitations. First, the correction for right-truncation in equation (4) results in a non-linear function which may become intractable and is prone to convergence difficulties, especially when the between-cluster variance is substantial. Second, the motivating example considered only the impact of right-truncation on Poisson-distributed outcomes. However, the correction for truncation in equation (4) may be generalized to other settings to cater for other types of truncations (i.e. left, right and interval which is also referred to as double truncation) for the normal,^45^ Poisson, negative binomial^46^ and other families of truncated distributions.^47^ As such, there is a need to validate the results in those settings.

In summary, CRTs are an important component of the evidence required to support the introduction of many interventions against infectious diseases, in which the unit of intervention is the community rather than the individual. In order to ensure that adequate statistical power for CRTs is maintained, the presence of right-truncation on count outcomes should be accounted for. Moreover, subsequent analysis of the trial data should account for right-truncation to ensure that the parameter estimates obtained are consistent, to facilitate correct inferences.

## Supplementary Data

Supplementary data are available at *IJE* online.

## Funding

This work was supported by grants from the Bill and Melinda Gates Foundation (L.M.M. and A.C.G.) and the Innovative Vector Control Consortium (K.J.F. and A.C.G.). We additionally acknowledge Centre support from the UK Medical Research Council and Department for International Development.

## Supplementary Material

dyz277_Supplementary_Data
